# The autophagy protein Def8 is altered in Alzheimer's disease and Aβ42-expressing *Drosophila* brains

**DOI:** 10.1038/s41598-023-44203-6

**Published:** 2023-10-10

**Authors:** Sebastián Oyarce-Pezoa, Guilherme Gischkow Rucatti, Francisco Muñoz-Carvajal, Nicole Sanhueza, Wileidy Gomez, Sandra Espinoza, Mario Leiva, Nicolás García, Daniela P. Ponce, Carol D. SanMartín, Diego Rojas-Rivera, Natalia Salvadores, Maria I. Behrens, Ute Woehlbier, Melissa Calegaro-Nassif, Mario Sanhueza

**Affiliations:** 1 Center for Resilience, Adaptation and Mitigation, Universidad Mayor, Temuco, Chile; 2 Laboratory of Autophagy and Neuroprotection, Vicerrectoría de Investigación, Universidad Mayor, Santiago, Chile; 3 PhD Program in Biomedicine, Universidad de los Andes, Santiago, Chile; 4 Center for Biomedical Research and Innovation (CiiB), Universidad de los Andes, Santiago, Chile; 5 PhD Program in Neurobiology, Universidad Mayor, Santiago, Chile; 6 PhD Program in Integrative Genomics, Universidad Mayor, Santiago, Chile; 7 Centro de Investigación Clínica Aplicada (CICA), Hospital Clínico Universidad de Chile, Santiago, Chile; 8 Instituto de Nutrición y Tecnología de los Alimentos (INTA), Universidad de Chile, Santiago, Chile; 9 Departamento de Neurociencia, Facultad de Medicina, Universidad de Chile, Santiago, Chile; 10 Departamento de Neurología y Psiquiatría, Clínica Alemana de Santiago, Santiago, Chile; 11 Departamento de Neurología y Neurocirugía, Hospital Clínico Universidad de Chile, Santiago, Chile; 12 Center for Integrative Biology, Universidad Mayor, Santiago, Chile; 13 Escuela de Biotecnología, Facultad de Ciencias, Universidad Mayor, Santiago, Chile; 14 Escuela de Tecnología Médica, Facultad de Medicina y Ciencias de la Salud, Universidad Mayor, Santiago, Chile; 15 Escuela de Medicina, Facultad de Medicina y Ciencias de la Salud, Universidad Mayor, Temuco, Chile

**Keywords:** Neuroscience, Diseases

## Abstract

Alzheimer's disease (AD) is the most common neurodegenerative disorder, characterized by protein accumulation in the brain as a main neuropathological hallmark. Among them, Aβ42 peptides tend to aggregate and create oligomers and plaques. Macroautophagy, a form of autophagy characterized by a double-membrane vesicle, plays a crucial role in maintaining neuronal homeostasis by degrading protein aggregates and dysfunctional organelles as a quality control process. Recently, DEF8, a relatively uncharacterized protein, has been proposed as a participant in vesicular traffic and autophagy pathways. We have reported increased DEF8 levels in lymphocytes from mild cognitive impairment (MCI) and early-stage AD patients and a neuronal profile in a murine transgenic AD model. Here, we analyzed DEF8 localization and levels in the postmortem frontal cortex of AD patients, finding increased levels compared to healthy controls. To evaluate the potential function of DEF8 in the nervous system, we performed an in silico assessment of its expression and network profiles, followed by an in vivo evaluation of a neuronal *Def8* deficient model using a *Drosophila melanogaster* model of AD based on Aβ42 expression. Our findings show that DEF8 is an essential protein for maintaining cellular homeostasis in the nervous system, and it is upregulated under stress conditions generated by Aβ42 aggregation. This study suggests DEF8 as a novel actor in the physiopathology of AD, and its exploration may lead to new treatment avenues.

## Introduction

Alzheimer's disease (AD), the most common type of dementia, is characterized by the accumulation of intracellular deposits of hyperphosphorylated Tau called neurofibrillary tangles (NFT) and extracellular deposits of misfolded amyloid beta protein (Aβ)^1^. Aβ peptide is generated by the proteolytic processing of the amyloid precursor protein (APP), and its accumulation leads to the development of Aβ plaques^2,3^. Aβ species and Tau are substrates for lysosomal clearance, suggesting their accumulation might be due to these pathways' failure, including macroautophagy (herein named autophagy). Autophagy corresponds in volume to the most important cellular degradation mechanism, characterized by the formation of a double membrane vesicle (autophagosome) capable of sequestering protein aggregates and dysfunctional organelles for their degradation in the lysosome^4^. A decrease in autophagy activity during aging is associated with a decline in brain skills, including memory and motor ability, as shown in organism models such as mice and flies^5,6^.

Autophagy is a highly regulated process by several conserved evolutionary protein complexes^7^. The ULK1/2 protein kinase complex (Atg1 in yeast and Drosophila) initiates the formation of autophagosomes to elongate the vesicle through the Atg5 complex, Beclin1/Atg14L, and the Atg8 orthologs LC3/GABARAP^4^. The autophagosome maturation, regulated by Beclin1, UVRAG, Pacer, and inhibited by Rubicon, is followed by its fusion with the lysosome through the PLEKHM1/RAB7A/HOPS complex, giving rise to the degradation and subsequent recycling of the substrates^8–11^. In AD, a disruption in this process has been reported, including failures in the traffic and transport of autophagosomes to the lysosome region and a decrease in the degradative activity of lysosomes^12–14^. Consequently, vesicular bodies from the autophagy pathway have been reported to be increased in AD brain^13,15^. These events trigger an accumulation of protein aggregates and dysfunctional organelles within autophagosomes or intermediate vesicles, simultaneously generating extracellular Aβ aggregates^16,17^.

DEF8 (Differentially expressed in FDCP 8) is a small protein of 459 amino acids that shares the C-terminal region with a group of proteins^18^, including PACER, which antagonizes Rubicon, and PLEKHM1, all involved in endolysosomal and autophagy processes^8,9,18–20^. DEF8 stabilizes the PLEKHM1 and RAB7A complex in osteoclasts, regulating lysosomal positioning^21^. Recently, we reported that DEF8 presents a neuronal profile in the central nervous system (CNS) in a murine transgenic AD model and shows a differential expression in peripheral blood mononuclear cells (PBMC) from patients with MCI and early-stage AD^22^. Notably, in a recent study exploring the components of Lewy bodies (LB) in postmortem brain samples from Parkinson's disease patients, researchers found colocalization of DEF8 and α-synuclein, the most abundant component of LBs^23^. Overall, these data suggest a role for DEF8 in protein aggregate formation and/or turnover. However, the function of DEF8 in the complex formed with PLEKHM1 and RAB7A and its impact on the CNS are mainly uncharacterized. Here, we report an increase in DEF8 levels in postmortem brain samples from late-AD patients, showing a mainly neuronal profile. To characterize Def8 function in CNS homeostasis, we evaluated the presence of Def8 in the *Drosophila melanogaster* nervous system and explored in silico its conservation, expression, and interactions. Def8 downregulation revealed an essential function of this protein in maintaining cellular homeostasis in adult flies' nervous system, viability, and functional behavior. Furthermore, Def8 levels are increased in fly brains expressing Aβ42, suggesting a potential role for Def8 in the pathogenesis associated with protein aggregation in AD.

## Methods

### Human brain tissue samples

Paraffin-embedded samples from human postmortem brain tissues were a kind donation from the Knight AD Research Center (Knight ADRC) at Washington University (St. Louis, MO, USA) under the approved Institutional Ethical Review Board protocol (#T1011). The operational criteria for AD classification were applied using the consensus neuropathologic criteria for AD and non-AD disorders, defined by the National Alzheimer Coordinating Center (NACC)^24^. The Clinical Dementia Rating (CDR) was applied to determine dementia severity. Postmortem tissues used in this study included three healthy (non-demented) controls (CDR = 0) and three severe AD cases (CDR = 3) with 6 years of disease duration (Table 1).

**Table 1:** Identification and information of the human frontal cortex samples used for the immunocytochemical detection of DEF8.

Donor	Gender	Age of decease	Condition	Disease duration (years)	Comorbidities
1	Female	92.6	Healthy	–	Hypertension, osteoarthritis
2	Female	85.7	Healthy	–	Hypertension, depression, hernia repair
3	Female	90	Healthy	–	–
4	Female	95	AD	6	Osteoarthritis, arthritis, anemia, peripheral vascular disease, allergy
5	Male	54	AD	6	Depression
6	Female	98	AD	6	Hypertension

### Histology of human brain tissue samples

10 µm postmortem brain samples were analyzed using the procedures described in^24,25^. Briefly, the slides were deparaffinized with xylol and hydrated with descending alcohol concentrations until reaching distilled water. For IF, epitopes were exposed to citrate buffer at 96 °C for 1 h. Slides were washed three times with 0.05% Tween20 in PBS1X. After blocking non-specific binding sites with 3% BSA in PBS1X solution, slices were incubated with primary antibodies diluted in 3% BSA and 0.05% Tween20 in PBS1X solution overnight at 4 °C. Primary antibodies and dilutions were as follows: anti-DEF8 (Biorbyt, rabbit, 1:1000) and anti-NeuN (EMD Millipore Corp., mouse, 1:500). Secondary antibodies and dilutions: anti-mouse Alexa 555 (Thermo Fisher Scientific, 1:500), and anti-rabbit Alexa 488 (Thermo Fisher Scientific, 1:1000), prepared in a 3% BSA-0.05% Tween20 in PBS 1X solution with DAPI (Invitrogen, 1:10.000), incubated for 2 h. Slides were washed and mounted with Fluoromount-G (Invitrogen). For amyloid β (Aβ) deposition analysis, brain samples were exposed to 70% formic acid for 20 min. Blocking was performed with 3% BSA in PBS 1X Tween20 0.02% for 1 h. Primary antibody 4G8 (Biolegend, mouse, 1:1000) was incubated overnight at 4 °C. Alexa 555 secondary antibody (Thermo Fisher Scientific, mouse 1:1000) and DAPI (Thermo Fisher Scientific, Waltham, MA, USA 1:10,000) was employed as a nucleus marker incubated for 2 h. All samples were analyzed using an inverted microscope (Leica Dmi8), and quantification analysis was performed by the ImageJ software using 10× and 40× images. Images were taken with a Leica TCS SP8 confocal microscope with 63× objective magnification. ImageJ and LAS X software were used to process the stacked images. Colocalization analysis of DEF8 and NeuN in brain tissues was performed using the Imaris software (Bitplane). Briefly, confocal images were processed by applying the same thresholds for the two fluorophores (green for DEF8 and red for NeuN). The software provided a colocalization coefficient (Mander's), which was used comparatively.

### Def8 sequence conservation analysis

The National Center for Biotechnology Information (NCBI) databases were used to obtain the genomic sequences of the DEF8 genes in Humans (Gene ID: 54849) and *Drosophila melanogaster* (Gene ID: 39381). The UniProt database was used to obtain the amino acid sequences of the DEF8 proteins in humans (Uniport code Q6ZN54) and Drosophila (Uniport code Q9VTT9). The NCBI tools BLASTn and BLASTp were used to compare genomic and amino acid sequences, respectively, followed by the CD-search (Conserved Domain Search) tool to compare conserved domains within the sequences. Sequence alignment and 3D structural information were obtained with the platform PROMALS3D^26^.

### Def8 expression profiling

Expression levels of *Def8* (CG11534), *Plekhm1* (CG1166), and *Rab7* (CG5915) in *Drosophila melanogaster* were extracted from single-cell analysis platform Scope (http://scope.aertslab.org) using the Aerts_Fly_AdultBrain_Filtered_57k database filtered by age (0 and 50 days). Likewise, human *DEF8*, *PLEKHM1*, and *RAB7A* expression levels were similarly obtained from the Zhang_Human_Brain_30k datasets for early-stage (young) gene expression and from the Allen Institute for Brain Investigation for expression in stages of aging (elderly). Data was uploaded to Scope and analyzed using the counts per million (CPM) normalization delivered by the platforms^27^.

### Predicted interaction networks

To generate and analyze *Def8* gene interaction networks, the Cytoscape v3.8.0 software was used. The GeneMANIA application was used to generate gene interacting networks that predict genetic functions, identifying the genes most related to a set of genes of interest using an association by cause approach^28^, using *Drosophila* and *Homo sapiens* databases. The STRING software was used to visualize interaction networks associated with processes and pathologies, where the interaction networks of autophagy and AD in humans were extracted^29^. A network including the putative interactors of human DEF8 was generated using Ingenuity Pathway Analysis (IPA) (QIAGEN Inc., https://www.qiagenbioinformatics.com/products/ingenuity-pathway-analysis). Using the IPA Knowledge database, upstream and downstream regulators of DEF8, as well as pathway enrichment, were displayed.

### *Drosophila melanogaster* lines

Flies available in our laboratory and used here were obtained from the Bloomington (BL) Drosophila Stock Center at Indiana University. They include the wild type (WT) lines used as control Canton S and W1118; the Gal4 drivers elav-Gal4 (specific to the nervous system), GMR-Gal4 (specific to the eye), and Actin-Gal4 (ubiquitous expression); the transgenic lines UAS-Ctrl.Trip (BL#35787), as a control construct for RNAi expression from the TRiP collection^30^, UAS-Aβ42.E693G Arctic (BL#33774), capable of expressing human Aβ42 peptides with the Arctic modification E693G^31^; and UAS-CG11534 RNAi (BL#28312), capable of expressing RNAi directed against Def8, decreasing its expression n. All flies were maintained in a standard medium for Drosophila at 25 °C under 12/12 h of the darkness-light-light cycle.

### Survival assessment

To assess the lifespan of flies, food vials containing them were kept at 25 °C, and the number of dead flies was counted over time (n = 25 flies × 4 vials; three replicates per genotype). The flies' viability was monitored until the last individual in the population died under normal conditions exceeding 60 days.

### Motor activity assessment

Weekly tests were performed to understand how Def8 deficiency affects motor performance: 10 flies were placed in an empty plastic vial and gently tapped to knock the flies to the bottom. The number of flies climbing that crossed a laser beam (Drosophila Activity Monitor, Trikinetics) was scored for 10 s and 10 climbs. The data was collected, averaged, and represented as a percentage of climbs.

### Evaluation of neurodegeneration in the eye

Ocular morphology was evaluated to visualize potential cytotoxic effects in the Drosophila nervous system. Five days after the hatching of flies expressing the eye-specific promoter GMR-GAL4, eye images were obtained using a light microscope (Leica S9i Stereoscopic Microscope). For adult retinas, samples were processed as stated previously^32^. After removing external layers, retinas were fixed in 4% PFA and stained with phalloidin conjugated to Alexa Fluor 647 (Invitrogen). Rhabdomeres were manually quantified, and their mean per ommatidium was calculated for all images, where seven rhabdomeres are usually visible in a normal ommatidium.

### Dissection and visualization of larval and adult brains

Third-instar larvae and adult brains were dissected in PBS and fixed with 4% paraformaldehyde for 30 min. After washing with a solution of 0.1% Triton in PBS (PBT), samples were blocked with 10% normal goat serum (NGS) for 2 h at room temperature. Primary antibody staining was performed overnight at 4 °C: anti-DEF8 (Biorbyt, 1:500) and anti-Aβ 4G8 (Biolegend, 1:500) in 5% NGS solution. Secondary antibodies and DAPI were incubated at a concentration of 1:1000 in a 5% NGS solution for 1 h. Finally, samples were transferred to a Fluoromont-G slide, covered, and sealed using nail varnish to prevent drying.

### Evaluation of Def8 expression by qPCR

Total RNA was extracted from 30 fly heads using TriZol reagent extraction (Sigma-Aldrich). The RNA concentration was measured for each sample in duplicate using a spectrophotometer (Thermo ScientificTM MultiskanTM GO–Microplate Spectrophotometer). The purity of samples was evaluated using the absorption ratio of 260/280 nm. Reverse transcriptase PCR (RT-PCR) was carried out using the iScript RT kit for the synthesis of complementary DNA (cDNA) (BioRad), with a sample volume equal to 20 µL and a concentration of 250 ng/µL. The obtained cDNAs were used as a template for real-time PCR using Eva's green qPCR master mix (SolisBioDyne) on a StepOne Plus machine (Applied Biosystems). Primer sequences to target Def8 were designed as follows: Fw 5′ TACACGGGCGTTGCCCATT 3′, Rv 5′ TGAGTATCGCAAATCTACCAGGT 3′. 18S gene was employed as a housekeeping gene: Fw 5′ AGAAACGGCTACCACATCCA 3′, Rv 5′ CCCTCCAATGGATCCTCGTT 3′.

### Protein visualization by Western Blot

To evaluate and quantify proteins, 30 adult fly heads were used, flash-frozen with liquid nitrogen for 15 s, followed by a vortex for 5 s, ensuring the correct separation of the fly's head. The heads were then isolated and ground with the help of a plastic rod in a RIPA buffer solution with protease inhibitors. The mixture was centrifuged at 13,000 rpm for 15 min, and the supernatant was used for protein quantification using the BCA Protein Assay Kit protocol (Thermofisher PierceTM). Samples were run on SDS-PAGE gels and transferred to PVDF membranes by Trans-Blot TurboTM RTA Transfer Kit (BioRad). Primary antibodies used were anti-DEF8 (Biorbyt, #orb183031, 1:1000) and the anti-β-Actin loading control (ThermoFisher, #MA1-744, 1:3000). Secondary antibodies were used at a concentration of 1:5000 (Jackson Immuno Research). Membranes were visualized using ChemiDocTM MP (BioRad). Original images of each full-length blot are available in Supplemental data.

### Statistical analysis

The data are represented as the mean with its standard error (SE) in all graphs using the GraphPad Prism 9 software. For the comparison of the two groups, the T-Student test was used. The one-way ANOVA test was used to compare more than two groups. For the Tukey comparison, the Bonferroni process was used. A two-way ANOVA test was used to compare more than two variables, with Bonferroni correction for multiple comparisons. For survival analysis, the Log-Rank Test was used. P values less than 0.05 were considered significant.

## Results

### DEF8 levels are increased in the postmortem frontal cortex of Alzheimer's disease patients

**Figure 1:**
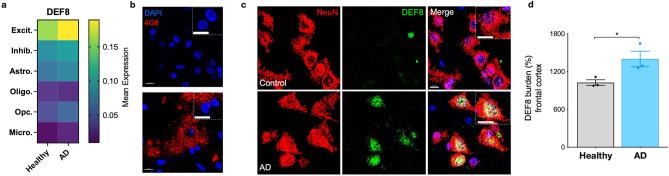
Immunohistochemical detection of DEF8 in frontal cortex sections of healthy subjects and AD patients. (**a**) Heat map of mean expression of DEF8 in each brain cell type: Exci. (Excitatory neurons), Inhib. (Inhibitory neurons), Astro. (astrocytes), Oligo. (oligodendrocytes), Opc. (oligodendrocyte progenitor cells), and Micro. (microglia) originated from Single-nucleus RNA-seq profiling of postmortem prefrontal cortex^33^. Postmortem samples from the frontal cortex from control (24 individuals no-AD) and AD patients (24 individuals). (**b**,**c**) Confocal Z-stack images of human frontal brain cortex sections in control (healthy) and AD patients. A magnification from the merged images is shown in a dashed square. Bar = 10 µm. (**b**) Immunostaining shows the detection of Aβ (red, 4G8 immunostaining) and nucleus (blue, Dapi). (**c**) DEF8 (green) and the neuron marker NeuN (red). Nuclei are stained with DAPI (blue). Images represent three AD patients and three healthy subjects. (**d**) DEF8 burden intensity fluorescence quantification in frontal cortex sections from healthy subjects and AD patients. Data are mean ± SEM (n = 3). *p < 0.05.

Previous results showed that DEF8 protein levels were increased in PBMC from MCI and early AD patients^22^. We also reported a neuronal expression profile of DEF8 in a transgenic AD model^24^. To explore the expression profile of DEF8 in the human CNS in the AD pathology context, we analyzed its expression in the frontal cortex from 24 controls and 24 AD patients from a dataset from single-nucleus RNA sequencing (snRNA-seq)^33^. As expected, DEF8 is abundantly expressed in neurons (excitatory and inhibitory neurons) compared to astrocytes, oligodendrocytes, oligodendrocytes progenitors, or microglia (Fig. 1a). Indeed, in AD tissues, DEF8 showed increased expression in excitatory neurons compared to controls (Fig. 1a). Transcriptional levels are not always correlative to protein levels. To evaluate if DEF8 protein levels are correlated with increased mRNA expression in AD tissues, we performed immunostaining of DEF8 in postmortem frontal cortex samples from AD patients and compared it to samples from control (healthy) individuals. First, we checked the Aβ plaques in both samples, finding a significant staining in AD patients compared to controls (Fig. 1b). Immunostaining of DEF8, along with the neuronal marker NeuN, was performed on brain tissue sections obtained from three severe AD patients and three control non-demented individuals (healthy), as illustrated in Fig. 1c. The analysis revealed a significant increase in protein staining in the AD condition (Fig. 1c,d), primarily localized in neurons, as shown in the merged image with NeuN (Fig. 1c, Supplementary Fig. 1). Our results show that protein levels of DEF8 significantly rise in human AD brain samples. Noteworthy, DEF8 is primarily located in cells positive to a neuronal marker.

### *CG11534* is the functional Drosophila ortholog of autophagy-related Def8

**Figure 2:**
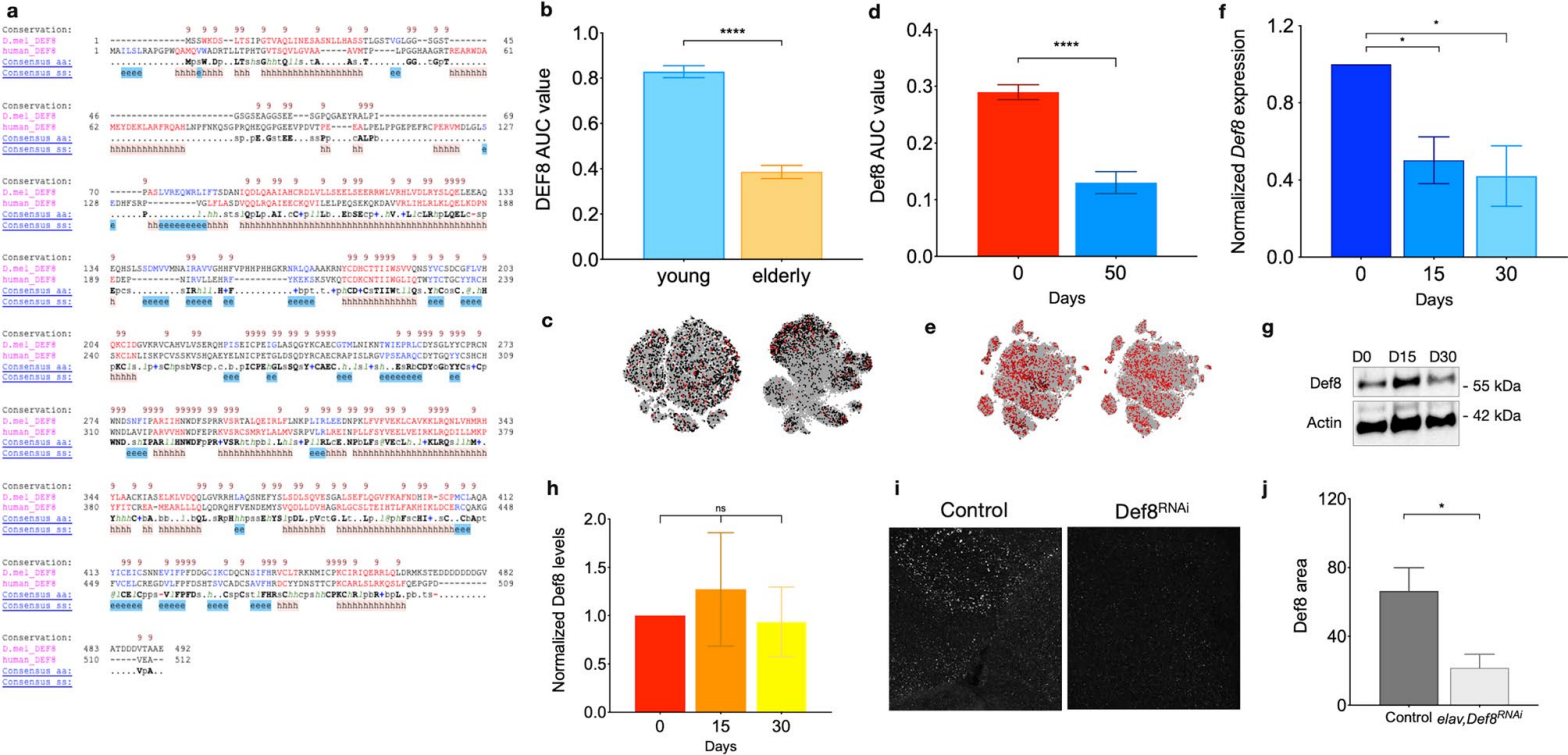
CG11534 is the *Drosophila melanogaster* homolog of human Def8. (**a**) Alignment of DEF8 in humans and CG11534 (Def8) in Drosophila, where the shared domains of C1_DEF8 (in green) and zf-RING_9 (in pink) are highlighted. Stars indicate conservation and dots similarity. E cutoff value = 0.01. (**b**) Conserved Domain Search results showing RUN domain and location in the amino acid sequence. (**c**–**f**) Def8 expression was acquired from in silico analyses. (**c**) Normalized expression levels of human *DEF8* and (**d**) Drosophila *Def8*, AUC value (area under the curve/area under the curve). Graphic representation of (**d**) human and (**f**) fly expression clusters obtained from the SCope platform. (**g**) *Def8* expression levels over time in adult fly brains. Bars represent standard error. *p < 0.05. (**h**) Representative western blot of Def8 expression in wild-type adult fly brains over time (**i**) and (**j**) its quantification of relative Def8 protein expression levels in fly heads (30 heads per sample; n = 3). Bars represent standard error, ns: p > 0.05; *p < 0.05; ****p < 0.0001.

While Def8 has been previously linked to the endolysosomal pathway in osteoclasts^21^, a specific mechanism and potential association with increased levels in AD brain samples remains unclear. To investigate the possible role of Def8 in AD progression, we sought to characterize this protein in flies. Initially, we determined the evolutionary conservation of Def8 by utilizing the DRSC Integrative Ortholog Prediction Tool (DIOPT). The stronger human Def8 ortholog in Drosophila was the previously uncharacterized fly gene *CG11534*. Both sequences showed 72.73% nucleotide homology and 39% aminoacidic homology (Fig. 2a). Using SCope, we found that Def8 is highly expressed in brain flies' excitatory, inhibitory, and cholinergic neurons (Supplementary Fig. 2). To explore the functional conservation of the protein, we corroborated that both sequences share the putative RH (Rubicon Homology) and C1_DEF8 domains, which carry a strong homology with other autophagy-related proteins (Fig. 2a). These data indicate a structural homology between both genes and their products in the different species. To compare the *DEF8* expression pattern in human and Drosophila CNS, we explored the Allen Institute for Brain Science (Fig. 2b,c) and SCope platforms, respectively (Fig. 2d,e). We found a decrease in DEF8 expression with aging in both species. We corroborated this observation by measuring *CG11534* expression in fly heads processed at 0-, 15- and 30-days post eclosion and demonstrating in vivo the same decreasing pattern in the DEF8 ortholog (Fig. 2f). Based on the predicted protein homology, we tested the immunoreactivity of the human-directed DEF8 antibody in total proteins isolated from adult fly heads. We observed that protein levels did not decrease with aging as with gene expression (Fig. 2g,h). Next, we generated flies expressing the RNAi against *CG11534* mRNA under the neuron-specific driver *elav-Gal4* and their controls. Neurons downregulating *CG11534* significantly reduced immunoreactivity to hDEF8ab compared to their age-matched controls (Fig. 2i,j), suggesting an effective model of downregulation in the nervous system and, importantly, that hDEF8ab recognizes the fly ortholog in neurons. All the previous data suggest a strong homology between DEF8 and its Drosophila ortholog CG11534, referred to from now on as Def8.

### Def8 is functionally linked to endolysosomal and autophagy pathways in neurons

**Figure 3:**
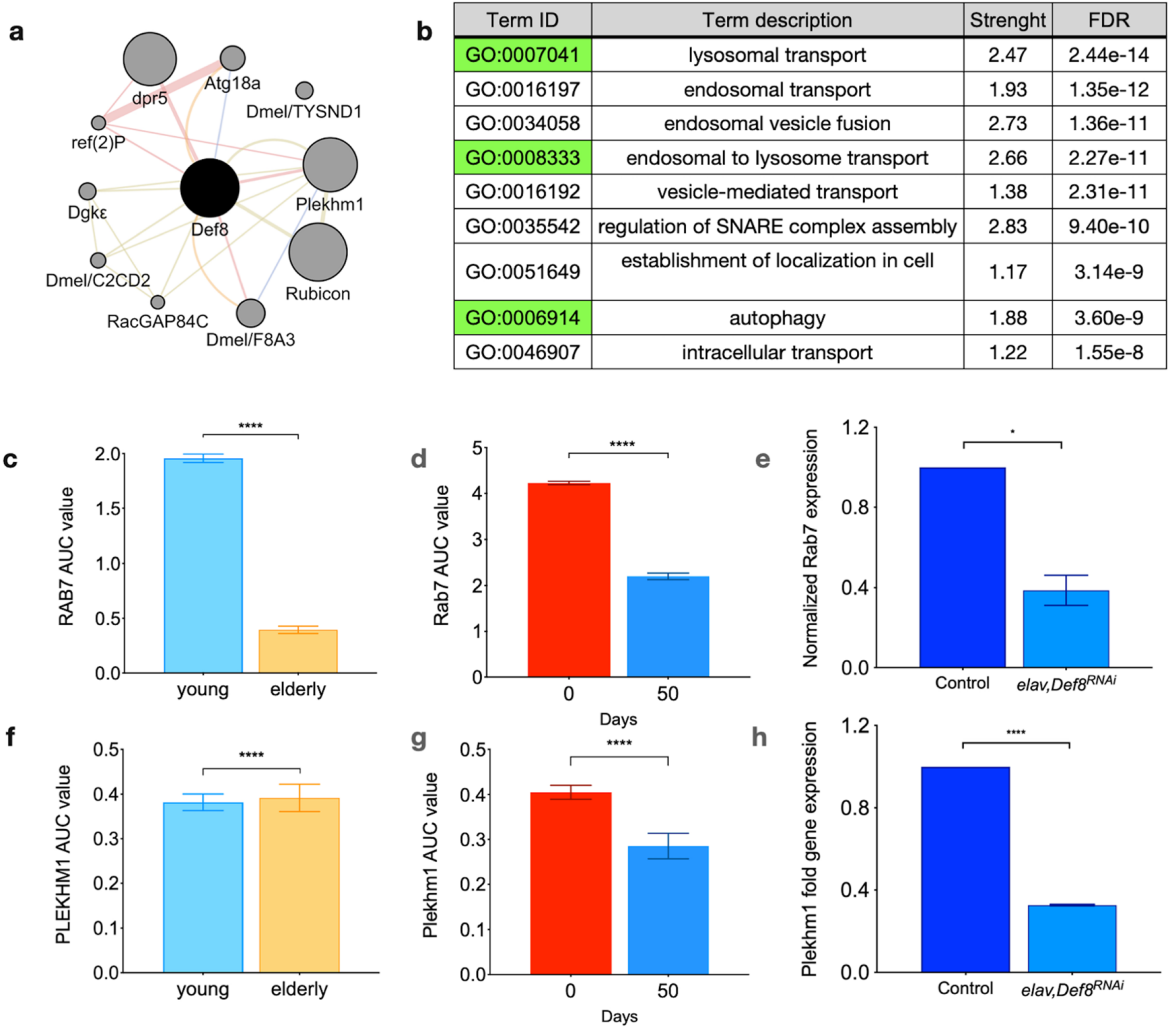
Def8 network in *Drosophila melanogaster* shows autophagy and endolysosomal-associated genes. (**a**) Def8 gene interaction network displaying protein interaction connection (pink), co-expression (light blue), shared domain (light brown), and prediction (orange). (**b**) List of Gene Ontology (GO) terms found for Def8 network. (**c**) Expression of human *RAB7A*, (**d**) Drosophila *Rab7* from in silico studies in different lifetimes (**e**) and *Rab7* expression from wild-type and Def8 mutant larval brains by real-time quantitative PCR. (**f**) Expression of human *PLEKHM1*, (**g**) Drosophila *Plekhm1* from in silico studies in different lifetimes, and (**h**) *Plekhm1* from wild-type and Def8 mutant larval brains by real-time quantitative PCR. *p < 0.05; ****p < 0.0001.

To explore a potential functional conservation of Def8, we queried its interaction network in flies on the platform GeneMania^28^ (Fig. 3a). Def8 interacts in Drosophila with Rubicon and CG6613 (herein called Plekhm1), an ortholog of human PLEKHM1, as observed in humans (Supplementary Fig. 2 and Supplementary Tables 1 and 2). Plekhm1 and Rubicon are among the proteins sharing structural domains with Def8^18^, suggesting a putative common function in the autophagosome-lysosome fusion stage. Another known gene from the autophagic pathway member linked to the Def8 network in flies is Refractory to Sigma P (*ref(2)P*) (Fig. 3a), the ortholog of the selective autophagy receptor SQSTM1/p62^34^. Next, we performed a Gene Ontology (GO) term analysis and found that endosomal and autophagy-related terms were some of the most enriched categories of the Def8-interacting network (Fig. 3b). To explore whether Plekhm1 and Rab7, the ortholog of human RAB7A, shared the Def8 expression pattern in flies, we used the SCope software for an in silico analysis. We found that *Rab7* (Fig. 3c,d) and *Plekhm1* (Fig. 3f,g) decreased their expression with aging in the human and fly neurons in silico, as observed for Def8. We then inquired whether there was any change in *Rab7* or *Plekhm1* expression in the background of Def8 downregulation. We found that both genes were decreased compared to their controls (Fig. 3e,h), suggesting a functional correlation between these three genes.

**Figure 4:**
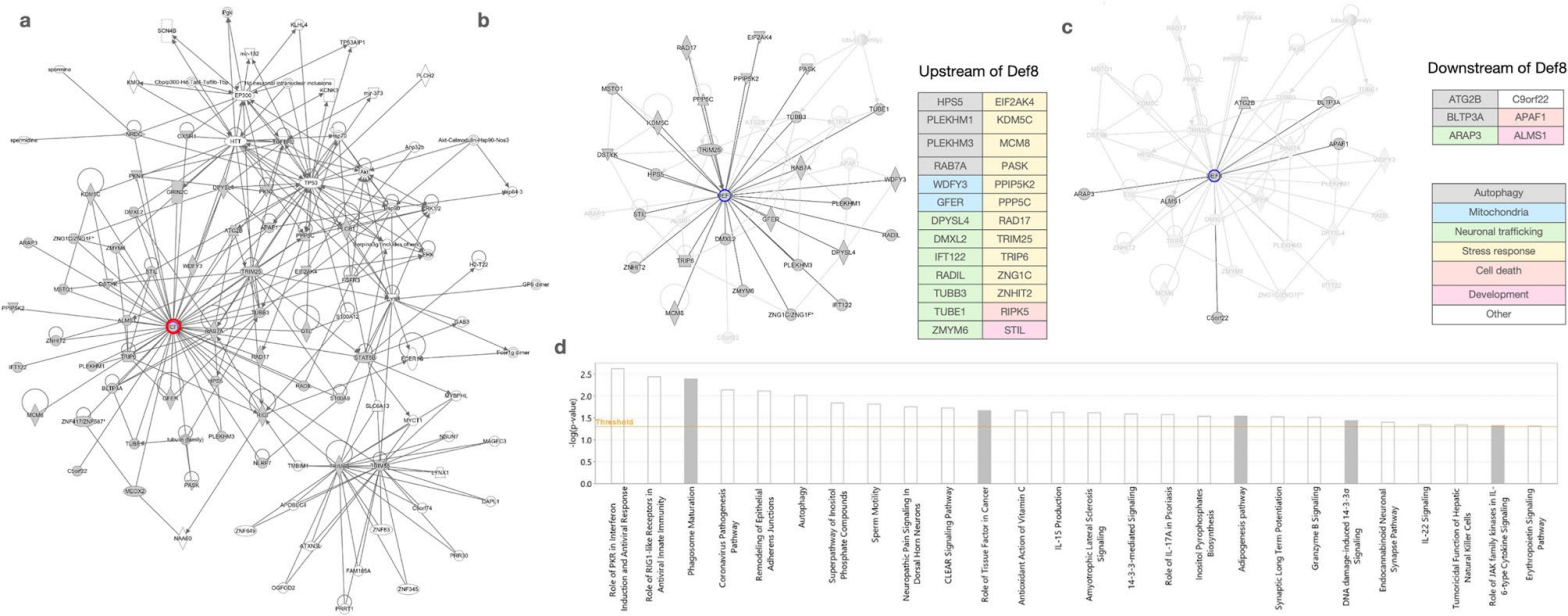
The Def8 Interactome. (**a**) Ingenuity pathway analysis (IPA) network generated with mammalian DEF8 interactors. Def8 is highlighted in red. IPA network for the upstream (**b**) and downstream (**c**) DEF8 interactors highlights the most relevant functional categories linked to the Def8 interactome. (**d**) Functional categories overrepresented in the Def8 interactome. Enrichment significance is expressed as the log10 corrected p-value.

To further explore the Def8 interaction network, we expanded our previous in silico analyses using the data platform Ingenuity Pathway Analysis (IPA). We queried available mammalian data (human, mouse, rat) in the IPA Knowledge database for DEF8 protein interactors. We identified two high-confidence networks (Supplementary Fig. 3 and Supplementary Table 3) that were then merged using exclusively interacting proteins into the Def8 interactome (Fig. 4a). In this last network, we observed previously identified interactors such as PLEKHM1 and RAB7A and recognized three main nodes: DEF8 itself, the tumor suppressor TP53, and the E3-Ubiquitin protein ligase TRIM63. This network participants' most enriched canonical pathways are linked to autophagosome maturation and antiviral response, suggesting a potential role for DEF8 in these pathways. The interacting proteins acting up- (Fig. 4b) or downstream (Fig. 4c) of DEF8 are mainly involved in autophagy, neuronal trafficking, and stress response. Furthermore, Neurological disease is one of the most enriched categories (p-value 5.55E^−4^–2.27E^−6^) linked to DEF8 interactome as predicted by IPA (Fig. 4d). Finally, the most relevant upstream regulator of the interactome was predicted by IPA as HNF4A (p-value 3.13E^−6^), an important transcriptional regulator previously linked to autophagy and metabolic disorders^35,36^. These bioinformatic analyses suggest that DEF8 has a role in the autophagy pathway and may have a role in metabolic and neurological disorders.

### Def8 expression in the nervous system is necessary for fly maintenance

**Figure 5:**
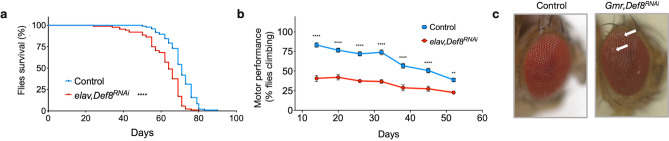
Degenerative phenotypes of Def8 downregulation in the nervous system of *Drosophila*. (**a**) Lifespan, as measured by the percentage of control flies (blue line) and mutant Def8 flies (red line) alive over time (n = 100 flies). (**b**) Motor performance as measured by climbing activity on control flies (blue line) and mutant Def8 flies (red line) over time (n = 15 flies, n = 10). (**c**) Eye morphology in Def8-deficient models. Stereomicroscope images of external eye structure from flies downregulating Def8 and their controls under the regulation of the eye-specific driver GMR-Gal4. White arrows indicate altered morphology. ****p < 0.0001, **p < 0.01.

To explore the functional consequences of the lack of Def8 in the Drosophila nervous system, we assessed the lifespan and motor performance of flies downregulating Def8 under the control of *elav-Gal4*. Compared with controls, mutant flies showed significantly reduced survival since day 40 after eclosion (Fig. 5a). Def8 downregulation also triggered a progressive age-dependent locomotion failure as measured by their climbing assay (Fig. 5b). Interestingly, worse motor performance in mutant flies was noticeable even at initial measured points, indicating a functionality deficit in the nervous system from the moment of fly eclosion and suggesting a developmental disturbance in neurons lacking Def8. To further explore a neurodegenerative scenario at early stages, we decreased Def8 expression specifically in the fly eye using the *GMR-Gal4* driver and phenotypically analyzed flies five days after eclosion. We noticed the presence of necrotic patches in flies lacking Def8, with no changes in eye size, suggesting a slight cytotoxic effect generated by Def8 knockdown at the neuronal level throughout development (Fig. 5c). This data indicates that Def8 is functionally necessary for Drosophila nervous system development and activity.

### Def8 is progressively upregulated in an Aβ-based AD model

**Figure 6:**
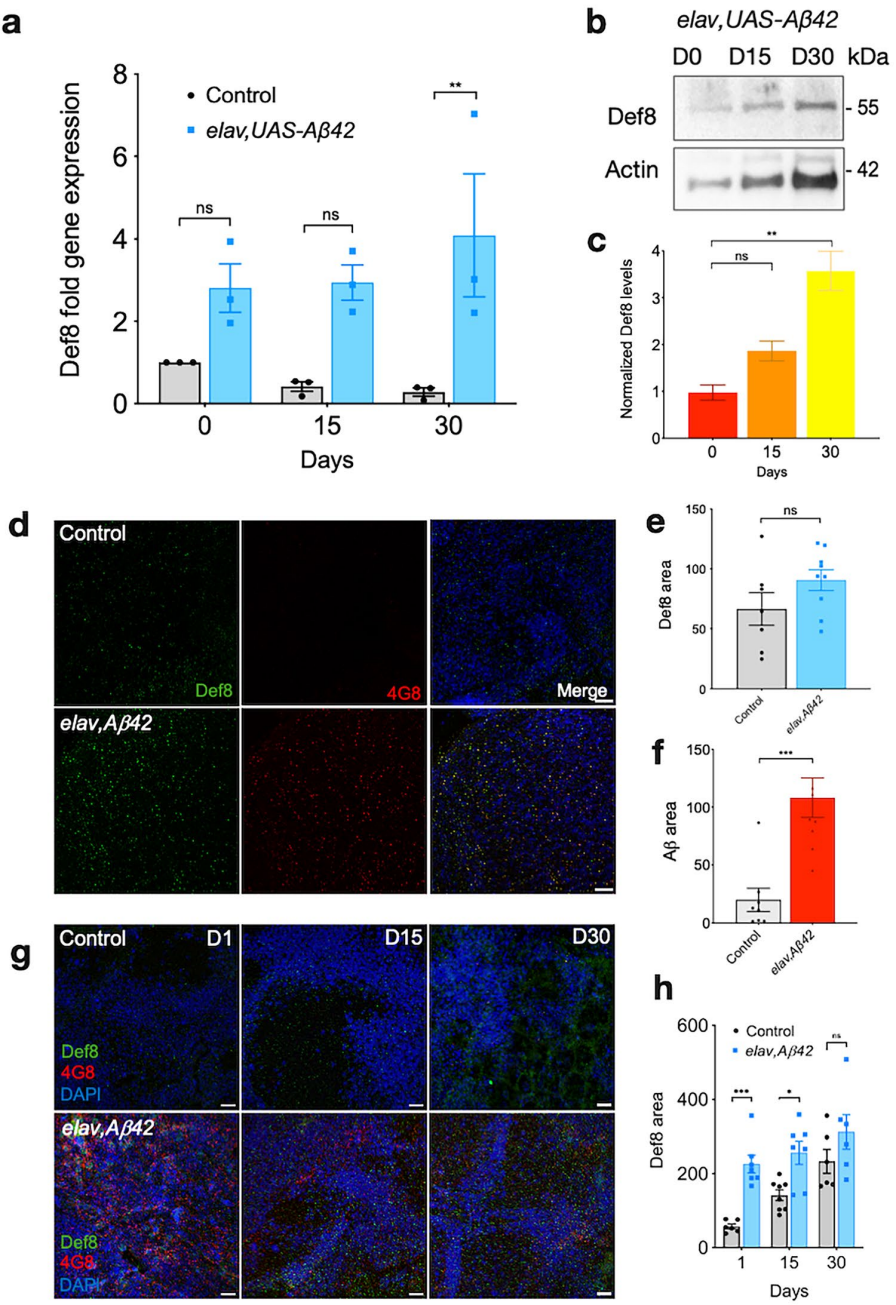
Def8 levels in the Drosophila Alzheimer's disease model. (**a**) Normalized *Def8* transcriptional expression levels over time in adult brains expressing Aβ42 (blue bars) and their control (grey bars). (**b**,**c**) Def8 protein levels in the adult fly brains expressing Aβ42 over time by western blot (**b**) and its quantification (**c**) in heads from *elav-Gal4, UAS-A*β*42* flies. (**d**) Immunofluorescence of *elav-Gal4, UAS-A*β*42* larval brains labeled with markers for Def8 (green) and Aβ42 aggregates (4G8, red). Differences in the Def8 (**e**) and Aβ (**f**) immunoreactivity per area between both genotypes (µm^2^). (**g**) Immunofluorescence of *elav-Gal4, UAS-A*β*42* adult brains at 1, 15, and 30 days post eclosion, labeled with Def8 (green) and Aβ42 oligomers (4G8, red). Differences in the Def8 (**h**) immunoreactivity per age between both genotypes. Data are mean ± SEM ***p < 0.001, **p < 0.01, ns: p > 0.005. Bar = 38 µm.

Def8 plays a role in autophagy linking Rab7-mediated endocytic trafficking, a mechanism conserved in *Drosophila* and mammals. A potential scenario for Def8's relevance for neuronal functionality and morphology is its role in aggregated protein clearance in the nervous system, a pathological hallmark of neurodegenerative diseases such as AD. To test this alternative, we studied the role of Def8 downregulation on the background of amyloid pathology, using a previously characterized AD fly model based on the expression of the human Aβ42 peptide in the nervous system^2^. First, we measured *Def8* expression in fly brains and found that its levels were increased in brains expressing Aβ42 compared to age-matched controls (Fig. 6a). Furthermore, *Def8* expression progressively increased with aging in Aβ42 flies as opposed to the age-dependent *Def8* decrease observed in wild-type brains (Fig. 6a). These results were consistent with the Def8 protein levels observed, as the stress condition caused by Aβ42 aggregates also triggered higher Def8 levels that increased with aging, reaching almost fourfold in older 30-day-old flies compared to recently eclosed mutant flies and more than fourfold levels found in aged mutant flies (30 days) compared to same-age control flies (Fig. 6b,c).

To further explore Def8 distribution and levels in Aβ42-expressing fly brains, we performed immunofluorescence analyses using Def8- and Aβ42-specific antibodies on larval brains and brains from 1, 15, and 30 days after the eclosion of flies. The analysis of the mushroom bodies of Aβ42-expressing larvae, structures in the *Drosophila* brain that are essential for olfactory learning and memory^37^, revealed that levels of Def8 were not significantly different from controls in this region, even though a significant difference was observed for the Aβ42 positive signal compared to control in larvae (Fig. 6d–f). However, when we analyzed Def8 immunoreactivity in adult brains, we observed a strong difference between both genotypes 1 day after eclosion (Fig. 6g,h). The significant difference is still visible at 15 days after eclosion, but the increase of Def8 signal in control flies progressively reduces that difference as observed at 30 days after eclosion (Fig. 6g,h). Nevertheless, we can confirm an increased Def8 immunoreactivity in adult flies expressing Aβ42 in their nervous system. To explore the role of Def8 on Aβ42 aggregation, we dissected brains from larvae co-expressing Aβ42 and Def8 RNAi in the nervous system. We did not detect a significant difference in Aβ42 aggregation compared to their control due to Def8-RNAi expression on larvae (Supplementary Fig. 5a,b). Also, no retinal structure deterioration was evident when quantifying the number of rhabdomeres per ommatidia in flies co-expressing Aβ42 and Def8-RNAi (Supplementary Fig. 5c,d). Altogether, our data suggest that Def8 is upregulated on the pathogenic background of Aβ42-expressing brains and that these levels further increase with aging.

## Discussion

Neurons depend on appropriate response mechanisms to cope with altered redox-signaling, accumulation of dysfunctional organelles and proteins, and high rates of synaptic transmission. Intracellular mechanisms that sustain functional neurons include autophagic clearance of accumulated cargos, highly efficient endocytic recycling, and active axonal retrograde transport of these vesicles through the neuron. Failure to form, transport, or degrade these vesicles has been proposed as drivers of neurodegenerative diseases, including AD^15,38–40^. The decrease in autophagy activity during aging contributes to a general reduction in neuronal homeostasis, contributing to the prevalence of neurodegenerative diseases^41,42^.

Although proteins from the autophagy machinery were first described in yeast^7^, they have several homologs in *C. elegans*, *Drosophila*^43^, and mammalian^4^. Here, we characterized the Def8 ortholog in *Drosophila*. Human and *Drosophila* proteins highlighted domain and sequence conservation, with Def8 positive immunoreactivity in flies, which allowed us to establish a structural homology in both species. Previous reports confirmed a strong link between Def8 and other autophagy components, Rab7a and Plekhm1^21,44^. Our network analyses also confirmed those observations in *Drosophila*. We found Def8 interactions with relevant proteins within the fly network, such as the complex fusion component Plekhm1, the selective autophagy receptor ref(2)P (SQSTM1/p62), and the autophagosome-maturation inhibitor Rubicon. The GO terms linked to the Def8-rooted network, "endolysosomal transport" and "autophagy," provide clues regarding the Def8 function and potential interaction partners. Interestingly, we observed the same expression patterns in fly brains over time as in humans for autophagy-related proteins *Rab7a*, *Plekhm1*, and *Def8*. Our data indicate that Def8 is functionally necessary for Drosophila nervous system development and activity, potentially mediated by interacting with Rab7 and Plekhm1 or other proteins from endolysosomal and autophagy pathways.

Here, we found *DEF8* transcriptional levels reduced in normal human and fly brains at different times, which could contribute to aging-associated diseases. DEF8 transcriptional expression from an RNA-seq dataset^33^ showed a marked neuronal expression, especially in excitatory neurons, compared to non-neuronal cells in human brains (astrocytes, oligodendrocytes, or microglia). In AD frontal cortex brain tissues, DEF8 maintains this expression pattern, increasing expression in excitatory neurons. We confirmed these data at protein levels in Alzheimer's postmortem brain cortex tissues. Interestingly, in a study in PD postmortem tissues, specifically sustancia nigra pars compacta, a vulnerable brain region associated with neurodegeneration in PD, DEF8 levels are increased^23^. Also, DEF8 was present with alpha-synuclein, a protein misfolded and aggregated in synucleinopathies such as PD, in insoluble fractions of SNps in patients^23^. This data reinforces the DEF8 potential role in proteostasis in neurodegenerative diseases.

Further analyses will be needed to determine whether increased DEF8 levels in pathogenic scenarios are due to compensatory mechanisms, representing neuron cells more resistant to neurodegeneration processes, or a late congested endolysosomal pathway accumulating DEF8-positive vesicles. Our phenotypic analyses showed, on the other hand, an active Def8 role in the *Drosophila* nervous system, as flies downregulating this gene in neurons compromised their lifespan, motor performance, and eye morphology. This data, with decreased expression during aging, suggests a functional role of the Def8-associated complex in neuronal maintenance. This effect would be especially relevant at the late stages of the fly life cycle, where more autophagic activity is necessary due to the increasing protein misfolding in neurons, even considering the significant reduction in the expression of their components.

Maintenance of the autophagy activity has been indicated to have anti-aging effects, as shown by the lifespan extension in mice ubiquitously overexpressing Atg5 or the specific neuronal overexpression of Atg9 or Atg8^45–47^. On the other hand, Rubicon loss of function in neurons improved lifespan in female flies^48^, which confirms the importance of autophagy activity to CNS since Rubicon is one of the primary inhibitors of the autophagy machinery. Notably, we recently reported increased Rubicon levels in postmortem brain tissues from AD patients, which could help explain a patient's predisposition to the disease^25^. Hence, the progressive decline in Def8 brain levels during aging in flies and humans and the detrimental effects under its neuronal downregulation could contribute to the aging predisposition to neurodegenerative disorders by affecting homeostatic routes such as autophagy.

As limited information is available in the literature from DEF8, we explored potential interactors by bioinformatics. In the *Drosophila* Def8 network, we found Def8 interacting with Plekhm1 and Rubicon, two proteins sharing the c-termini region with Def8 and the selective autophagy receptor ref(2)P. Ref(2)P is the mammal selective autophagy receptor SQSTM1/p62 homolog. This protein contains binding domains to ubiquitinated proteins (UBA) and the LC3 in the autophagosome membranes, acting as a bridge between the autophagy substrate and vesicle. The context of neurodegenerative disorders represents a drastic challenge for autophagic machinery. Here, we unveiled a strong upregulation of Def8 in fly brains expressing the human amyloidogenic protein Aβ42. It will be interesting to study the specific nature of this upregulation further, mainly whether a common regulatory mechanism shared with other autophagy components could be chemically upregulated. Even though we measured a significant increase in RNA and protein levels, we did not observe a positive Def8 immunoreactivity in larval brains, suggesting that another regulatory level may be shared with other autophagy-linked proteins, such as the interaction with Rab7 and Plekhm1. Further dissection of this data will determine Def8's role in the autophagy pathway and its modulation of AD pathogenesis, opening a novel target for neuroprotective and therapeutic approaches for the disease.

### Supplementary Information

Supplementary Information 1.

Supplementary Information 2.

## Data Availability

The datasets used and/or analyzed during the current study are available from the corresponding authors upon reasonable request.
