# Supplementary material for: The autophagy protein Def8 is altered in Alzheimer's disease and Aβ42-expressing Drosophila brains
Source: Sci Rep. 2023 Oct 10;13:17137. doi: 10.1038/s41598-023-44203-6 (PMC10564863; doi:10.1038/s41598-023-44203-6)
Supplement: Supplementary file 1 — Supplementary Information 1. [file 41598_2023_44203_MOESM1_ESM.docx]

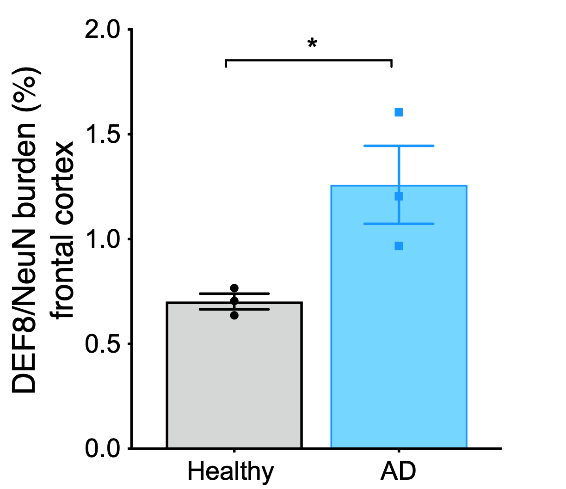


**Supplementary Figure 1.** DEF8 burden intensity fluorescence in frontal cortex sections normalized by NeuN (neuronal marker) intensity from healthy subjects and AD patients normalized by NeuN. Data are mean ± SEM (n=3). *p<0.05.


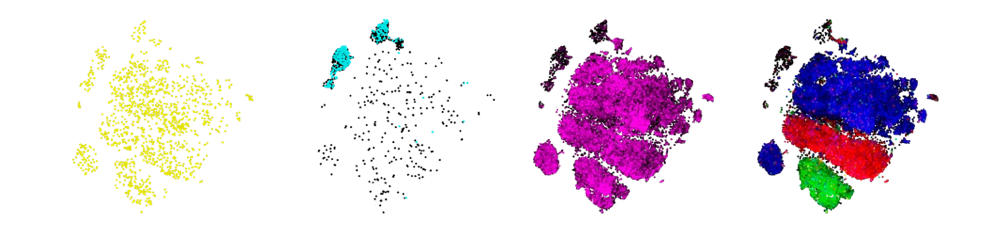


**Supplementary Figure 2. Images from Scope Single Cell Atlas of the coexpression of Def8 with markers for different cell types in Drosophila adult brain.** Yellow, whole brain Def8 expression; magenta, Repo>Glia; purple, Elav>Pan; neuronal; blue, VACht>Cholinergic neurons; red, VGlut1>Glutamatergic neurons, and green, Gad1>GABAergic neurons.


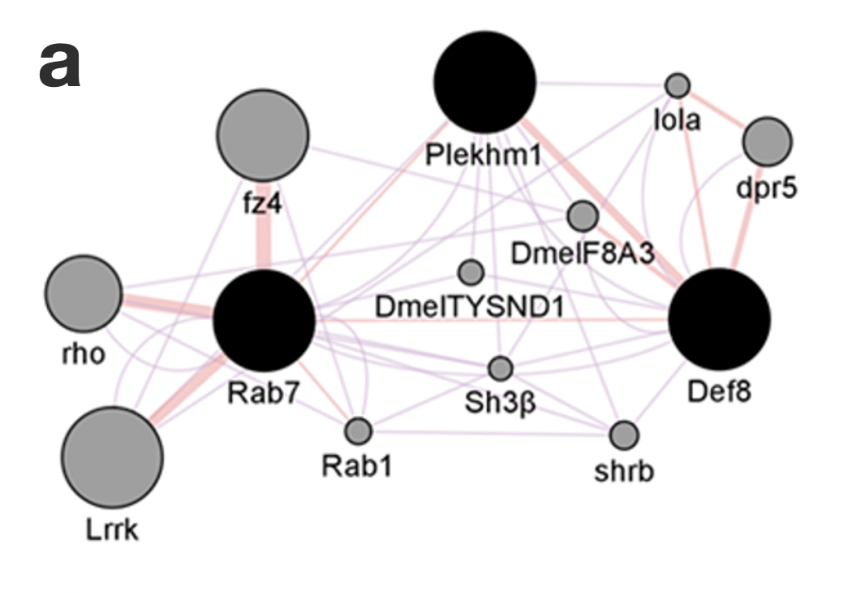

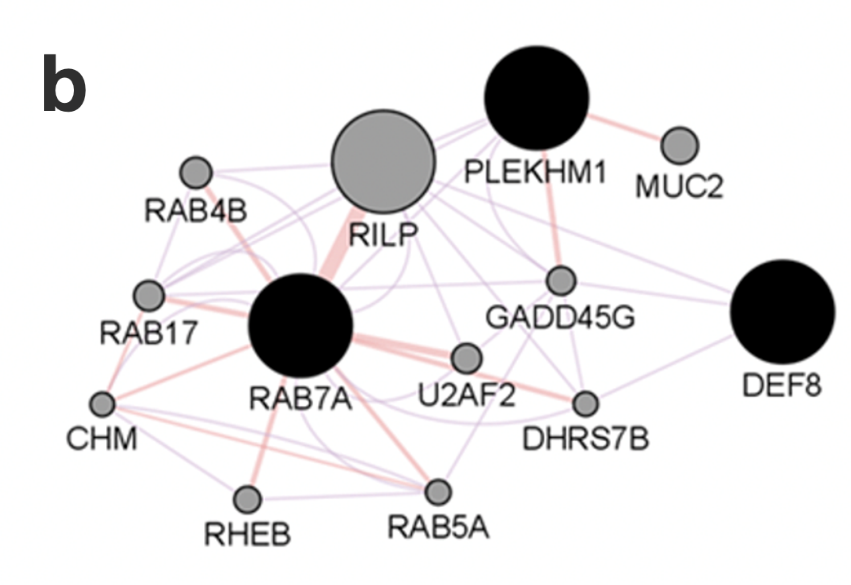


**Supplemental** **Figure 3. Relevant interaction networks in flies and humans. (a)** Interaction networks for the *Def8*, *Plekhm1*, *Rab7 Drosophila* genes. (**b)** Interaction networks for the *DEF8*, *PLEKHM1*, and *RAB7A* human genes. Pink: protein interaction; blue: coexpression; black: interest genes.

**Supplemental Table 1.** Gene ontology (GO) terms the most enriched processes from the network *Def8*, *Plekhm1*, and *Rab7* in Drosophila. FDR: False discovery rate.

| **Term ID​** | **Term description​** | **Strength ​** | **FDR​** |
| --- | --- | --- | --- |
| GO:0007041​ | Lysosomal transport | 2.47​ | 2.44e-14​ |
| GO:0016197​ | endosomal transport ​ | 1.93​ | 1.35e-12​ |
| GO:0034058​ | endosomal vesicle fusion​ | 2.73​ | 1.36e-11​ |
| GO:0008333​ | endosome to lysosome transport | 2.66​ | 2.27e-11​ |
| GO:0016192​ | vesicle-mediated transport​ | 1.38​ | 2.31e-11​ |
| GO:0035542​ | regulation of SNARE complex assembly​ | 2.83​ | 9.40e-10​ |
| GO:0051649​ | establishment of localization in cell​ | 1.17​ | 3.14e-9​ |
| GO:0006914​ | autophagy​ | 1.88​ | 3.60e-9​ |
| GO:0046907​ | intracellular transport ​ | 1.22​ | 1.55e-8​ |

**Supplemental Table 2.** Gene ontology (GO) terms the most enriched processes from the network DEF8, *PLEKHM1*, and *RAB7A* in humans. FDR: False discovery rate.

| **Term ID​** | **Term description​** | **Strength ​** | **FDR​** |
| --- | --- | --- | --- |
| GO:0007034 | vacuolar transport | 1.93 | 2.03e-10​ |
| GO:0007041​ | lysosomal transport | 1.94​ | 3.77e-07 |
| GO:0008333 | endosome to lysosome transport | 2.16 ​ | 1.29e-06​ |
| GO:0015031​ | ​protein transport | 1.01​ | 6.66e-06 |
| GO:0071702​ | organic substance transport​ | 0.88​ | 6.66e-06 |
| GO:0033036​ | macromolecule localization​ | 0.83​ | 1.59e-05 |
| GO:0006623​ | protein targeting to vacuole | 2.22 | 1.84e-05​ |
| GO:0034058​ | endosomal vesicle fusion​ | 2.75 | 5.07e-905 |
| GO:0006886​ | intracellular protein transport ​ | 1.08​ | 6.09e-05​ |


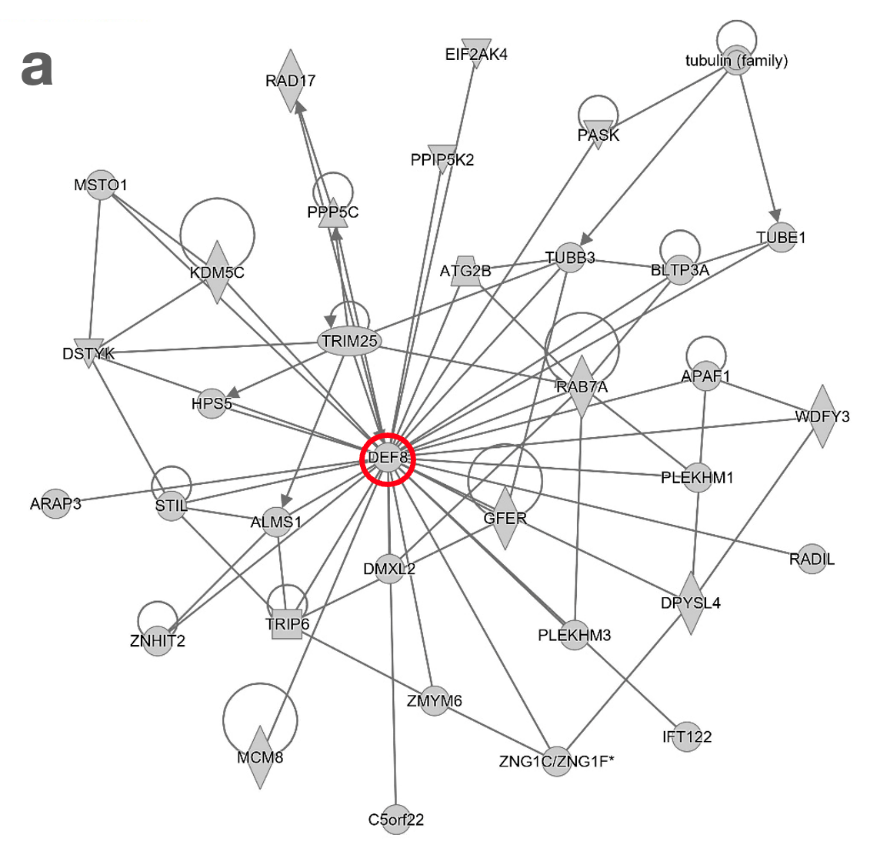

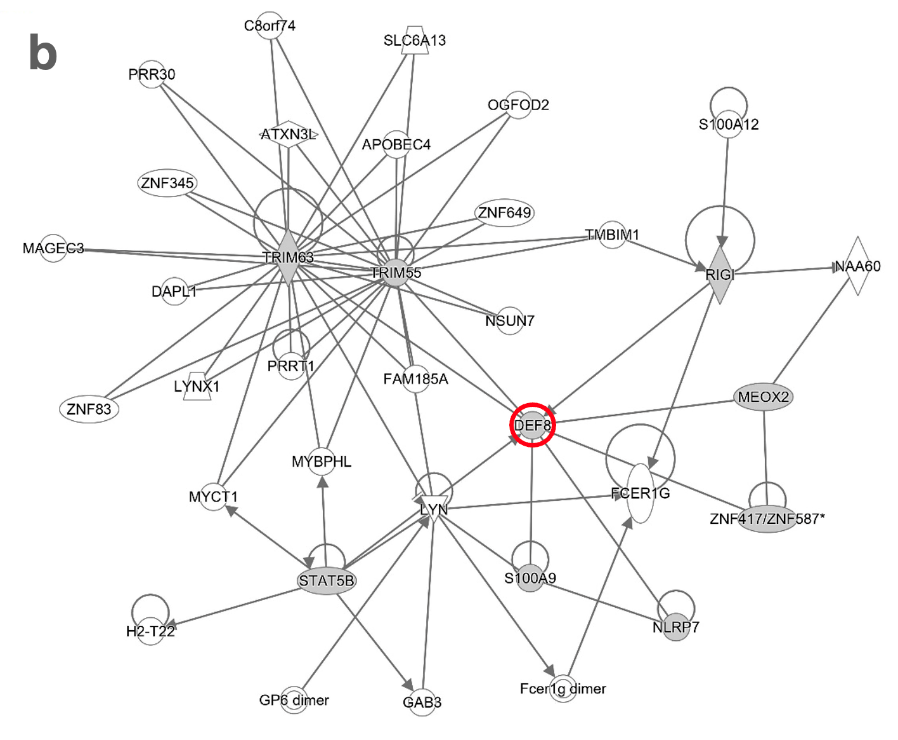


**Supplemental** **Figure 4.** Components for the ingenuity pathway analysis (IPA) networks generated with mammalian DEF8 interactors**. (a, b)** IPA networks obtained from DEF8 interactors. DEF8 is highlighted in red.

**Supplemental Table 3.** Components for the ingenuity pathway analysis (IPA) networks generated with mammalian DEF8 interactors. Top diseases and network scores are calculated to reflect high confidence levels for both networks.

| **Network** | **Top Diseases and Functions** | **Score** | **Focus Molecules** | **Molecules in Network** |
| --- | --- | --- | --- | --- |
| 1 | (Cancer, Hematological Disease, Immunological Disease) | 100 | 34 | ALMS1, APAF1, ARAP3, ATG2B, BLTP3A, C5ORF22, **DEF8**, DMXL2, DPYSL4, DSTYK, EIF2AK4, GFER, HPS5, IFT122, KDM5C, MCM8, MSTO1, PASK, PLEKHM1, PLEKHM3, PPIP5K2, PPP5C, RAB7A, RAD17, RADIL, STIL, TRIM25, TRIP6, TUBB3, TUBE1, tubulin(family), WDFY3, ZMYM6, ZNG1C/ZNG1F, ZNHIT2 |
| 2 | (Cell Death and Survival, Cellular Compromise, Neurological Disease) | 18 | 9 | AKT, AKT-Calmodulin-Hsp90-Nos3, Anp32b, Cbp/p300-Hd-Taf4-Taf9b-Tbp, **DEF8**, DTL, EP300, ERK, ERK1/2, FGFR3, GRIN2C, Hd-neuronal intranuclear inclusions, Hsp70, Hsp84-3, Hsp90, HTT, KCNK3, KLHL4, KMO, mir-132, mir-373, NRDC, OXSR1, Pgk, PKN2, PKN3, PLCB1, PLCH2, SCN4B, Serpina3g (9nclude others), spermidine, TGFBR2, TP53, TP53AIP1 |


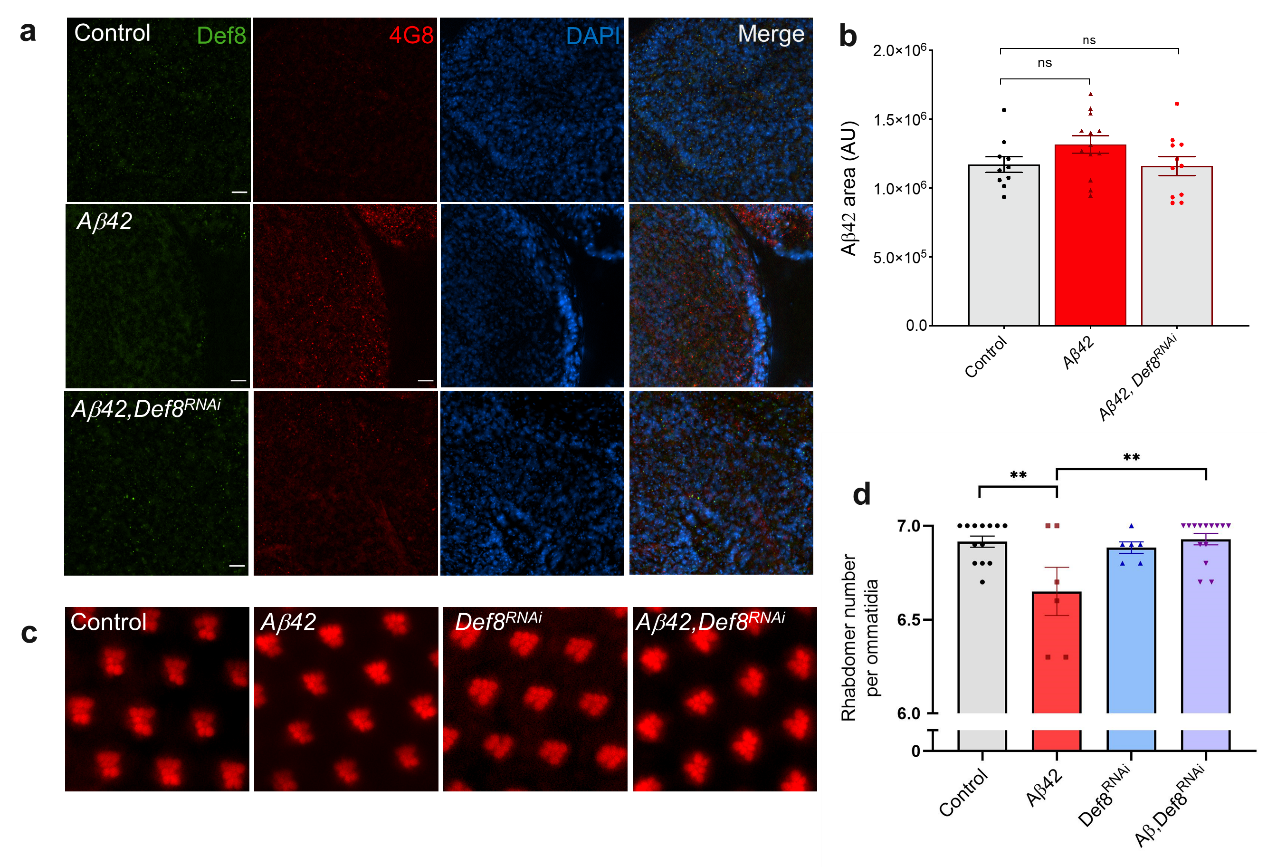


**Supplementary Figure 5.** Aβ42 accumulation in the *Drosophila* nervous system. (a) Immunofluorescence of *elav-Gal4* (Control), *elav-Gal4;UAS-Aβ42* (Aβ42), *elav-Gal4; UAS-Aβ42; UAS-Def8-RNAi* (Aβ42;Def8RNAi) larval brains labeled for Def8 (green) and Aβ42 oligomers (4G8, red). (b) Aβ immunoreactivity quantified for these three genotypes as stained area per 100 mm. (c) Adult retinas at 20 days post eclosion stained with phalloidin in red. (d) Quantification of the average rhabdomer number per ommatidia within each dissected retina. **: p<0.01. Bar = 10 µm.
