# Supplementary material for: The autophagy protein Def8 is altered in Alzheimer's disease and Aβ42-expressing Drosophila brains
Source: Sci Rep. 2023 Oct 10;13:17137. doi: 10.1038/s41598-023-44203-6 (PMC10564863; doi:10.1038/s41598-023-44203-6)
Supplement: Supplementary file 2 — Supplementary Information 2. [file 41598_2023_44203_MOESM2_ESM.pptx]

## Slide 1
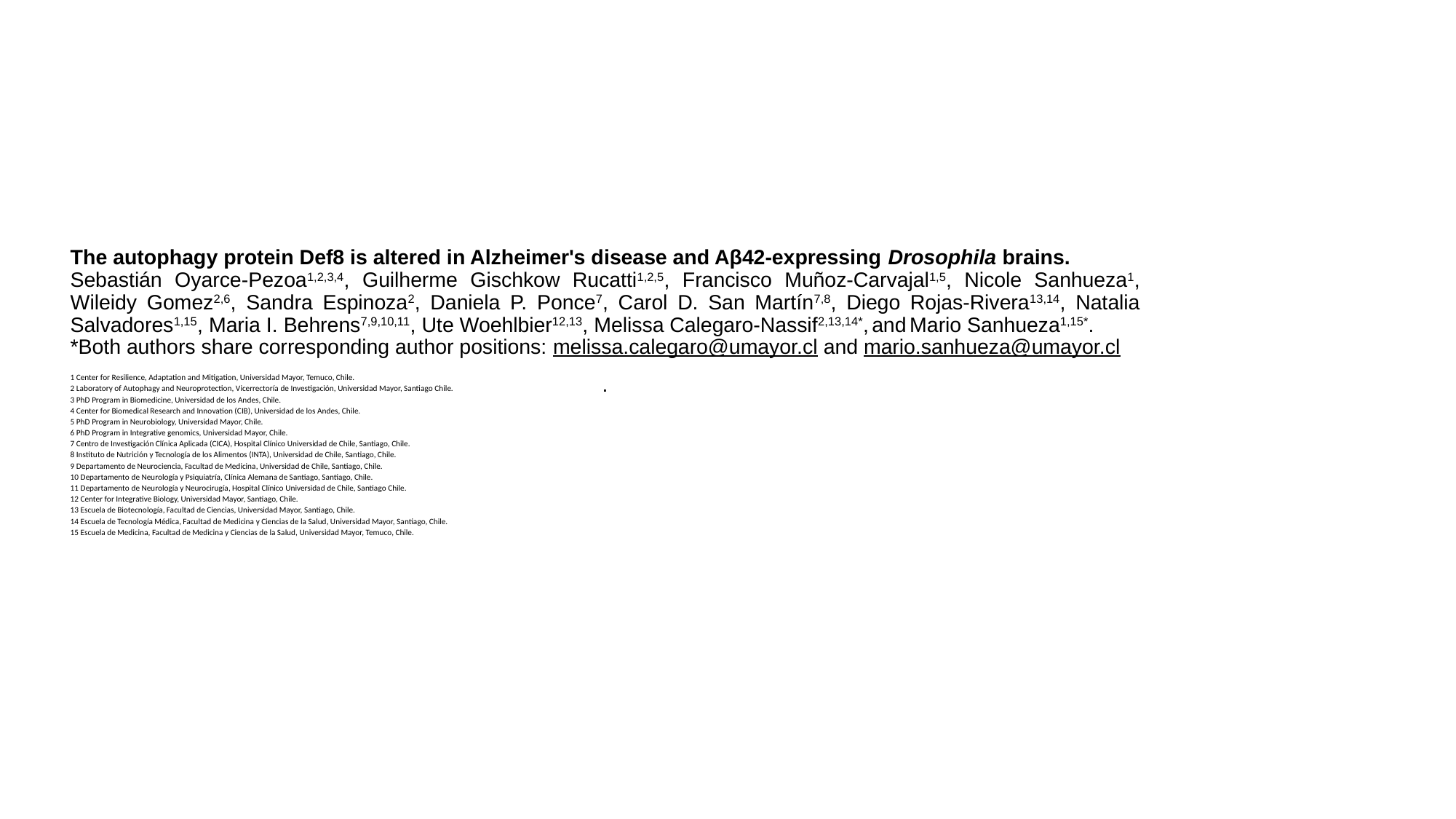

# The autophagy protein Def8 is altered in Alzheimer's disease and Aβ42-expressing Drosophila brains.
Sebastián Oyarce-Pezoa1,2,3,4, Guilherme Gischkow Rucatti1,2,5, Francisco Muñoz-Carvajal1,5, Nicole Sanhueza1, Wileidy Gomez2,6, Sandra Espinoza2, Daniela P. Ponce7, Carol D. San Martín7,8, Diego Rojas-Rivera13,14, Natalia Salvadores1,15, Maria I. Behrens7,9,10,11, Ute Woehlbier12,13, Melissa Calegaro-Nassif2,13,14*, and Mario Sanhueza1,15*.
*Both authors share corresponding author positions: melissa.calegaro@umayor.cl and mario.sanhueza@umayor.cl
.
1 Center for Resilience, Adaptation and Mitigation, Universidad Mayor, Temuco, Chile.
2 Laboratory of Autophagy and Neuroprotection, Vicerrectoría de Investigación, Universidad Mayor, Santiago Chile.
3 PhD Program in Biomedicine, Universidad de los Andes, Chile.
4 Center for Biomedical Research and Innovation (CIB), Universidad de los Andes, Chile.
5 PhD Program in Neurobiology, Universidad Mayor, Chile.
6 PhD Program in Integrative genomics, Universidad Mayor, Chile.
7 Centro de Investigación Clínica Aplicada (CICA), Hospital Clínico Universidad de Chile, Santiago, Chile.
8 Instituto de Nutrición y Tecnología de los Alimentos (INTA), Universidad de Chile, Santiago, Chile.
9 Departamento de Neurociencia, Facultad de Medicina, Universidad de Chile, Santiago, Chile.
10 Departamento de Neurología y Psiquiatría, Clínica Alemana de Santiago, Santiago, Chile.
11 Departamento de Neurología y Neurocirugía, Hospital Clínico Universidad de Chile, Santiago Chile.
12 Center for Integrative Biology, Universidad Mayor, Santiago, Chile.
13 Escuela de Biotecnología, Facultad de Ciencias, Universidad Mayor, Santiago, Chile.
14 Escuela de Tecnología Médica, Facultad de Medicina y Ciencias de la Salud, Universidad Mayor, Santiago, Chile.
15 Escuela de Medicina, Facultad de Medicina y Ciencias de la Salud, Universidad Mayor, Temuco, Chile.

## Slide 2
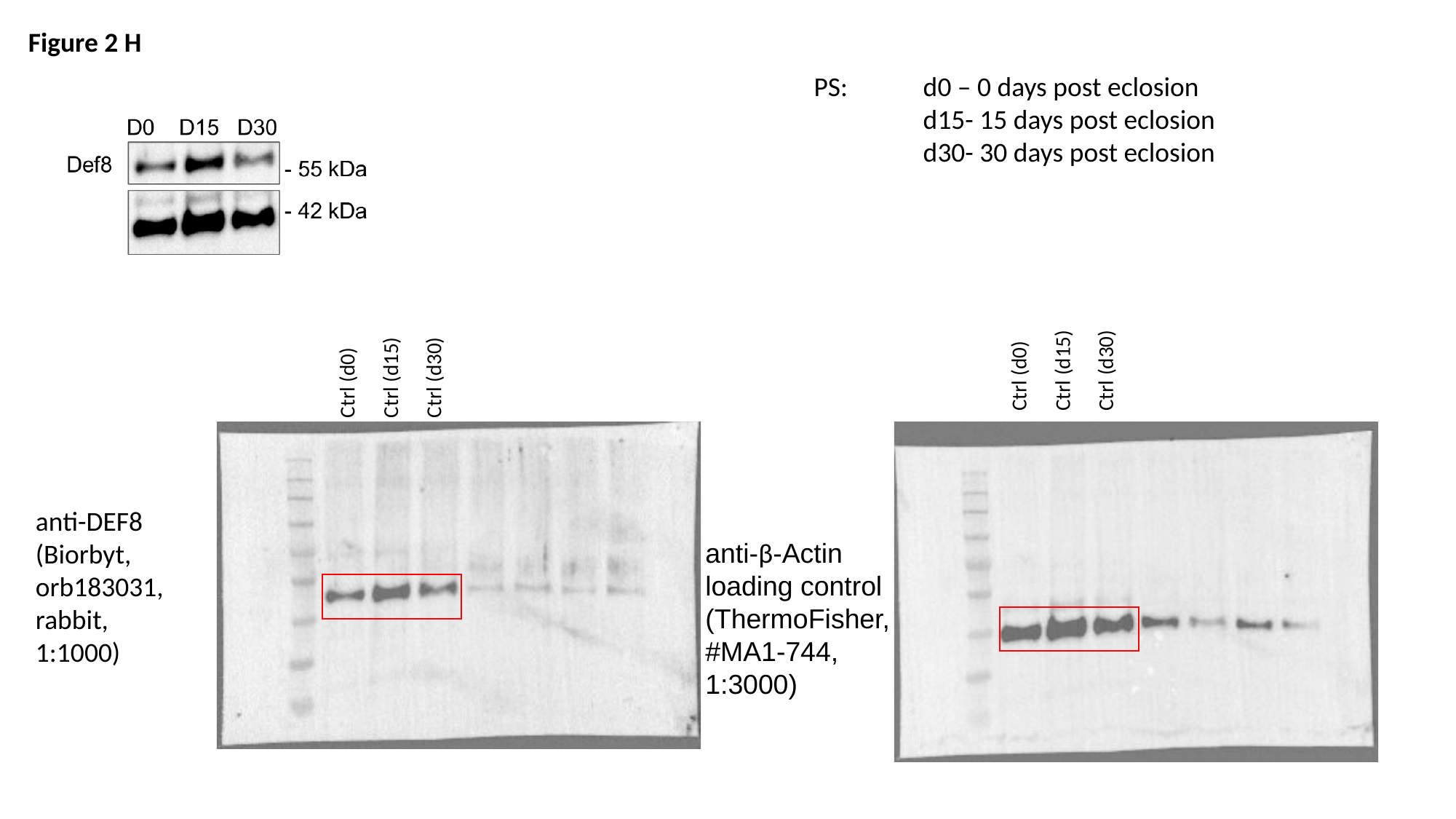

Figure 2 H
PS: 	d0 – 0 days post eclosion
	d15- 15 days post eclosion
	d30- 30 days post eclosion
Ctrl (d30)
Ctrl (d15)
Ctrl (d0)
Ctrl (d30)
Ctrl (d15)
Ctrl (d0)
anti-DEF8 (Biorbyt, orb183031, rabbit, 1:1000)
anti-β-Actin loading control (ThermoFisher, #MA1-744, 1:3000)

## Slide 3
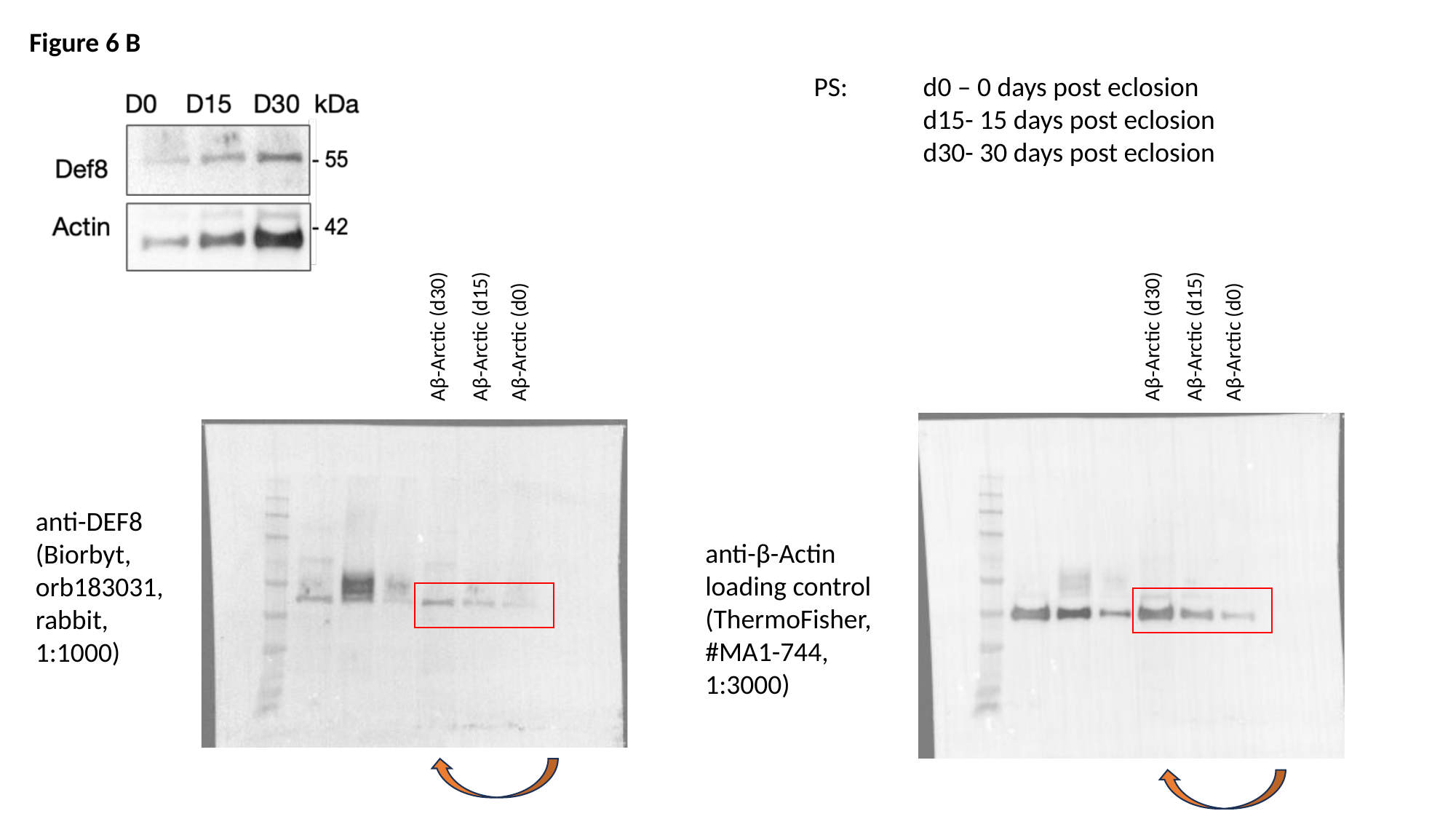

Figure 6 B
PS: 	d0 – 0 days post eclosion
	d15- 15 days post eclosion
	d30- 30 days post eclosion
Aβ-Arctic (d30)
Aβ-Arctic (d30)
Aβ-Arctic (d15)
Aβ-Arctic (d15)
Aβ-Arctic (d0)
Aβ-Arctic (d0)
anti-DEF8 (Biorbyt, orb183031, rabbit, 1:1000)
anti-β-Actin loading control (ThermoFisher, #MA1-744, 1:3000)
